# Global longitudinal strain: an early marker for cardiotoxicity in patients treated for breast cancer

**DOI:** 10.1007/s12471-022-01734-3

**Published:** 2022-11-26

**Authors:** D. van der Linde, I. van Hagen, K. Veen, H. Zuetenhorst, B. van Dalen

**Affiliations:** 1 Department of Cardiology, Franciscus Gasthuis & Vlietland, Rotterdam, The Netherlands; 2 Department of Cardiology, Thoraxcenter, Erasmus University Medical Centre, Rotterdam, The Netherlands; 3 Department of Epidemiology, Thoraxcenter, Erasmus University Medical Centre, Rotterdam, The Netherlands; 4 Department of Oncology, Franciscus Gasthuis & Vlietland, Rotterdam, The Netherlands

**Keywords:** Echocardiography, Ventricular ejection fraction, Cardiotoxic agents, Prevention

## Abstract

**Background:**

Patients treated with anthracyclines and trastuzumab are at increased risk of developing heart failure. Early diagnosis and treatment may prevent irreversible left ventricular (LV) dysfunction. This study investigates whether subclinical deterioration of global longitudinal strain (GLS) is a more reliable early predictor for LV dysfunction than three-dimensional (3D) LV ejection fraction (LVEF).

**Methods:**

Adult patients receiving anthracyclines and trastuzumab for breast cancer who had serial echocardiographic follow-up were included in this retrospective study. The primary endpoint was the necessity to temporarily pause chemo- or immunotherapy due to declining LVEF (decline in 3D LVEF of > 10 percentage points to < 53%). Linear mixed-effects models were used to assess the longitudinal evolution of 3D LVEF and GLS over time.

**Results:**

Fifty-one women were included, mean age 54 (50.5–57.6) years, with a total of 216 follow-up echocardiograms (mean follow-up 1.1 ± 0.45 years). GLS and 3D LVEF were significantly correlated (Spearman’s rho: −0.36, *p* < 0.001). A decrease in GLS significantly predicted a lower LVEF on the subsequent echocardiogram [ß −0.6, 95% confidence interval (CI) (−1.0 to −0.2), *p* < 0.006]. Conversely, prior LVEF did not significantly predict GLS on the subsequent echocardiogram [ß −0.04, 95% CI −0.1 to −0.01, *p* = 0.12]. Nine patients reached the primary endpoint. On average, patients who reached the primary endpoint had a relative decrease of 15% GLS at day 205 and an absolute 10% decrease of LVEF to LVEF < 53% at day 235.

**Discussion:**

GLS is able to identify subclinical LV dysfunction earlier than 3D LVEF measurement in women undergoing treatment for breast cancer with anthracyclines followed by trastuzumab.

**Supplementary Information:**

The online version of this article (10.1007/s12471-022-01734-3) contains supplementary material, which is available to authorized users.

## What’s new?

- Myocardial strain imaging using global longitudinal strain (GLS) is able to identify subclinical left ventricular (LV) dysfunction earlier than three-dimensional LV ejection fraction measurement in women undergoing treatment for breast cancer with anthracyclines followed by trastuzumab.
- It remains to be established whether the use of GLS to guide early cardioprotective therapy results in improved clinical outcomes and which patients are at highest risk of developing cardiotoxicity.

## Introduction

Cardio-oncology is an emerging field of interest in cardiology that focuses on the detection, monitoring and treatment of cardiovascular disease occurring as a side-effect of chemotherapy and radiotherapy. Cardiological assessment before starting potentially cardiotoxic cancer treatment is essential and should be continued throughout the oncological treatments, since left ventricular (LV) dysfunction can occur at any time.

The cardiotoxicity of anthracyclines is well-recognised, dose-related and irreversible [1]. Trastuzumab increases the cardiotoxicity of anthracycline treatment and can also cause cardiotoxicity when given on its own, but this cardiotoxicity is reversible [2]. When giving combined treatment with anthracyclines and trastuzumab, LV dysfunction has been reported in up to a third of the patients, with overt heart failure in up to 16% of patients [3].

A recent consensus paper from the European Society of Cardiology proposed a definition of chemotherapy-related cardiotoxicity as an LVEF decrease of > 10 percentage points from baseline to a value < 53% on repeat confirmatory echocardiographic imaging [4]. Nevertheless, routinely used two-dimensional (2D) and three-dimensional (3D) LV ejection fraction (LVEF) measurements may fail to detect subtle subclinical signs of LV dysfunction, which represent the first warning signs for cardiotoxicity. Once LVEF has deteriorated, it may be too late to reverse the anthracycline-induced cardiotoxicity. Monitoring the development of early subclinical cardiotoxicity may therefore be crucial; however, clear guidelines on how to do this are lacking.

Global longitudinal strain (GLS) has been found to be a useful marker to detect subclinical myocardial dysfunction [5, 6]. An early reduction in GLS of 10–15% during cancer therapy appears to be a useful parameter for the prediction of cardiotoxicity [4]. It has previously been shown that early deterioration of GLS can predict LVEF decline 3 months later [5]. GLS-guided introduction of cardioprotective medication been has shown to reduce the fall in LVEF at 1 year compared to standard LVEF guidance [7]. However, in these studies 2D echocardiography was used to assess LVEF, while 3D echocardiography has proven to be superior [8]. The added value of GLS over 3D LVEF is therefore unknown. Also, so far little is known about why some patients develop cardiotoxicity but others do not.

The primary objective of our study is to assess whether subclinical deterioration of GLS is a more reliable early predictor for LV dysfunction than 3D LVEF and can predict 3D LVEF decline later on. Furthermore, we will examine which baseline factors are associated with a high risk of cardiotoxicity in a cohort of women with breast cancer treated with anthracyclines followed by trastuzumab.

## Methods

### Patients and methods

Adult patients receiving anthracyclines and/or trastuzumab for breast cancer at Franciscus Gasthuis & Vlietland, Rotterdam, The Netherlands, from 2014 to 2017 and who had undergone echocardiography at baseline and every 3 months thereafter were identified. Exclusion criteria were an LVEF < 50% prior to chemotherapy or pregnancy. 2D echocardiographic image quality should be at least reasonable to allow reliable 3D LVEF assessment. Therefore, patients with moderate or poor image quality were excluded.

A retrospective chart review was conducted to collect chemotherapy regimens, treatment interruption data, baseline characteristics such as age, blood pressure, previous medical history and use of cardiovascular medications, and outcome data. The Cardiotoxicity Risk Score (CRS) proposed by the Mayo Clinic was calculated from baseline characteristics to estimate the a priori risk of developing cardiotoxicity [9].

LV function was measured by 2D (Simpson’s method) and 3D LVEF and myocardial strain analysis was done using 2D speckle-tracking imaging according to the recommendations of the European Society of Echocardiography [10, 11]. Echocardiography was performed using a commercially available ultrasound system (EPIQ7, Philips, Eindhoven, The Netherlands), equipped with a broadband (1–5 MHz) X5‑1 transducer. The datasets were exported to a TomTec server (4.7.0.3, TomTec Imaging Systems, Munich, Germany) for tracking analysis. Tracking quality was over-ridden in segments with fewer than two regions where the observer deemed tracking quality to be clearly acceptable. In cases with persistently more than two regions with inadequate tracking quality the echocardiogram was excluded from the analysis. GLS was calculated by averaging the 18 segmental values in the 2‑, 3‑ and 4‑chamber apical views. By convention, GLS results were interpreted as absolute values [11]. In other words, a change in GLS from, for example, −18% to −15% will be presented as a decrease in GLS.

The study was approved by the Medical Research Ethics Committee of the Rotterdam Hospital area. Due to the retrospective nature of the study design no informed consent was required. The study was conducted according to the principles of the 1975 Declaration of Helsinki.

### Endpoints

The endpoint of this study was the necessity to temporarily pause chemotherapy due to declining LVEF. Criteria for pausing treatment were: a decline in 3D LVEF of > 10 percentage points and a 3D LVEF of < 53% [12]. Secondary endpoints included all-cause mortality or a cardiac event, defined by New York Heart Association class II, III or IV heart failure or cardiac death.

### Statistics

Continuous data are presented as mean ± standard deviation (Gaussian distribution) or median and interquartile range (IQR) (non-Gaussian distribution). Categorical data are presented as frequencies (percentage).

Linear mixed-effects models were used to assess the longitudinal evolution of 3D LVEF and GLS and the absolute and relative percentage change of these outcomes compared to baseline over time. These models included random intercepts and slopes over time to capture the correlation of the repeated measurements in each patient. Natural splines with one knot were used to allow for flexibility of the subject-specific trajectories over time. Patients were censored at the time trastuzumab was paused.

Survival probabilities were estimated and visualised by the Kaplan-Meier method. Univariable Cox proportional hazard models were used to explore determinants associated with the endpoint.

The dropout of some patients is caused by LVEF itself, as this was the criterion for pausing treatment. Sensitivity analysis was performed, whereby the dynamic longitudinal evolution of GLS and 3D LVEF was inserted into a Cox model predicting dropout under the joint modelling framework. Modelling these entities together alleviates possible bias due to values that are missing not at random. The current value parameterisation was investigated [13].

## Results

In total, 51 females with a mean age at baseline of 54 ± 12 years were included in the study. After review of the echocardiograms, one patient was excluded due to inadequate quality of the echocardiographic imaging.

Baseline characteristics and echocardiographic characteristics at baseline are presented in Table S1 (Electronic Supplementary Material). In total, 12 (26%) patients had cardiac medications at baseline. During a mean follow-up duration of 1.1 ± 0.45 years, 5 patients died, yielding a Kaplan-Meier survival estimate of 70.5 ± 13.9% at 5 years. None of the deaths were related to a cardiovascular event. None of the patients showed signs of clinical heart failure or any cardiac event during follow-up.

### Longitudinal evolution of GLS and LVEF

In total, 216 follow-up 3D LVEF measurements were available (mean 4.5 per patient, range 1–6) and 215 GLS measurements (mean 4.5 per patient, range 1–6).

**Fig. 1:**
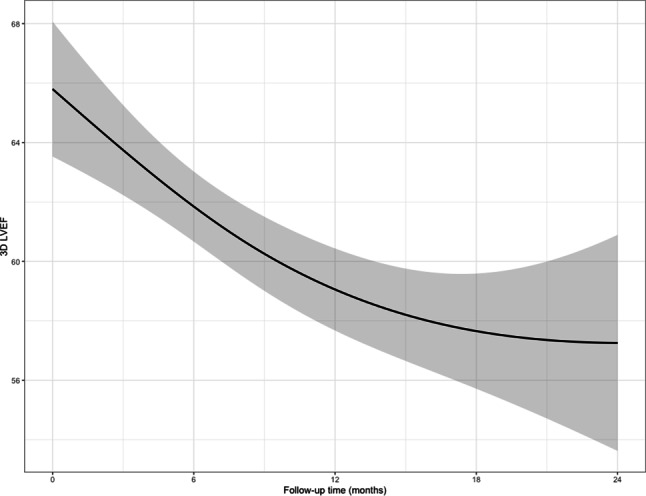
Kaplan-Meier curve of the longitudinal evolution of three-dimensional left ventricular ejection fraction (*3D LEVF*). *n* = 51

Initially, 3D LVEF decreased significantly over time [ß −14.7, 95% confidence interval (CI) −20.1 to −9.3, *p* < 0.001], whereas later in the follow-up period this decrease stagnated (β −4.7, 95% CI −12.7 to 3.3, *p* = 0.23), as estimated by a linear mixed model adjusted for baseline 3D LVEF. An effects plot of the modelled longitudinal evolution of 3D LVEF is presented in Fig. 1. The joint model showed little sensitivity in the estimates.

**Fig. 2:**
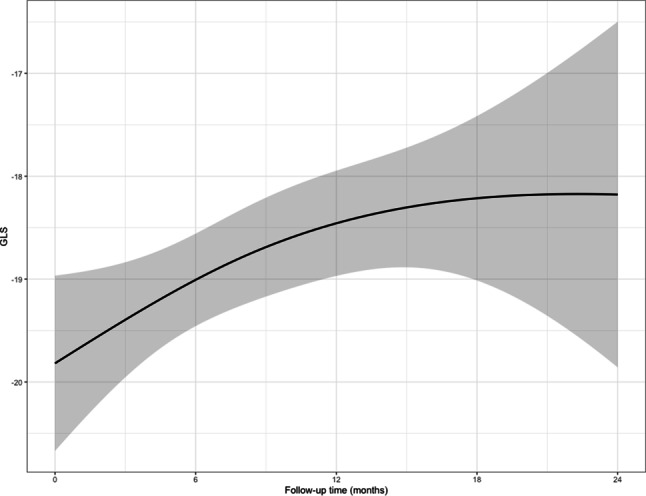
Kaplan-Meier curve of the longitudinal evolution of global longitudinal strain (*GLS*). *n* = 51

GLS initially decreased over time (β 2.7, 95% CI 0.8–4.6, *p* < 0.001), whereas later in the follow-up period this decrease stagnated (β 0.8, 95% CI −2.7 to 3.9, *p* = 0.58), as estimated by a linear mixed model adjusted for baseline GLS. An effects plot is shown in Fig. 2. Similar to 3D LVEF, the joint model showed little sensitivity in the estimates.

### Risk factors for longitudinal evolution of GLS and LVEF

The CRS was associated neither with a different starting point of LVEF and GLS, nor with different slopes over time (Figs. S1 and S2, Electronic Supplementary Material). Additionally, age and hypertension were not associated with different starting points and slopes over time.

### Pause in treatment

In none of the patients were anthracyclines paused for cardiac reasons. In 9 (18%) patients trastuzumab was paused due to a decline in 3D LVEF without clinical symptoms, on average for the duration of 36 days. This resulted in a freedom to pause trastuzumab of 59.4% ± 14.9% at 2 years (Kaplan-Meier estimate). The measurements of 3D LVEF and GLS in these 9 patients are shown in Fig. S3 (Electronic Supplementary Material). All patients with subclinical LV dysfunction were started on cardioprotective medication: 8 patients on angiotensin-receptor inhibitors and 1 patient on a beta-blocker. In all patients the decline in LVEF was transient. In 3 patients the oncologists chose not the restart the trastuzumab due to other side-effects (*n* = 1) or because the length of the therapy was considered adequate (*n* = 2).

CRS was not associated with a pause in trastuzumab treatment [hazard ratio (HR) 0.90, 95% CI 0.47–1.74, *p* = 0.77]. Additionally, age (HR 0.98, 95% CI 0.93–1.03, *p* = 0.46) and hypertension (HR 0.1.96, 95% CI 0.51–7.61, *p* = 0.33) were not associated with pausing trastuzumab. No attempts at multivariable modelling were conducted due to the limited number of events.

### GLS compared to 3D LVEF

GLS and 3D LVED were significantly correlated (Spearman’s rho −0.36, *p* < 0.001). A decrease in GLS significantly predicts a lower LVEF on the following echocardiogram (β −0.6, 95% CI −1.0 to −0.2, *p* < 0.006) (Fig. S4, Electronic Supplementary Material). Conversely, prior LVEF does not significantly predict GLS on the following echocardiogram (β −0.04, 95% CI −0.1 to −0.01, *p* = 0.12), suggesting that a decrease in GLS precedes an LVEF decline and not vice versa.

**Fig. 3:**
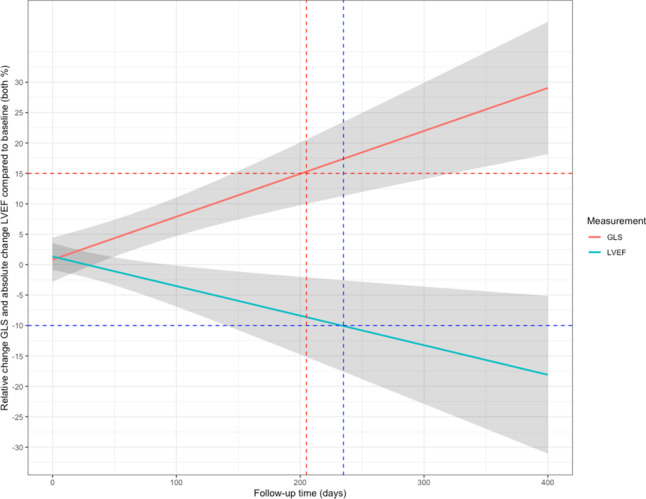
Effects plot of the longitudinal evolution of left ventricular ejection fraction (*LVEF*) and global longitudinal strain (*GLS*) in the nine patients that reached the endpoint. The *red dashed lines* show a worsening of GLS of +15% compared to baseline and its intercept with the *x*-axis. The *blue dashed lines* show a decrease in LVEF of −10% compared to baseline and its intercept with the *x*-axis

The relative and absolute percentage change compared to baseline of GLS and 3D LVEF were modelled in the 9 patients that reached the endpoint. On average, these patients had a relative decrease of 15% compared to baseline for GLS at day 205, 30 days earlier than the absolute decrease in LVEF of 10% to LVEF < 53% at day 235 (Fig. 3; Fig. S3, Electronic Supplementary Material).

## Discussion

While there are several reports in the literature about early detection of cardiotoxicity, ours is the first to compare 3D LVEF and GLS over time using a linear mixed-effects model over an extended follow-up period during trastuzumab therapy. We have demonstrated that GLS deteriorates earlier than 3D LVEF. Therefore, GLS may still be of added value regarding the detection of cardiotoxicity, even in the 3D echocardiography era.

The principal findings from this study indicate that: (1) subclinical LV dysfunction can be detected on average 30 days earlier by a GLS decrease than by a 3D LVEF decline; (2) no baseline characteristics, such as age, hypertension and CRS, could be identified as predictors for cardiotoxicity; (3) in our cohort none of the patients reached a clinical endpoint and LV dysfunction was reversible after pausing trastuzumab.

GLS is an established preclinical marker of cardiac dysfunction [4]. It is well known that GLS provides independent and incremental prognostic information regarding long-term risk of cardiovascular morbidity and mortality [14]. The longitudinal myocardial fibres responsible for longitudinal shortening seem particularly vulnerable at a relatively early stage to a wide range of cardiac diseases, including cardiotoxicity [7]. However, a 15% deterioration of GLS as a cutoff to be used in clinical practice will still be subject to discussion. The cutoff is based on small studies, and the predictive value with respect to heart failure is unknown [5]. In our cohort there were no clinical consequences, and it may be debated whether acting on this cutoff value, instead of acting on a subclinical drop in LVEF, adds to the long-term perspective of these patients.

In contrast to other studies, we were not able to demonstrate with our advanced statistical model that parameters such as increasing age, hypertension, 3D LVEF at baseline or CRS are clinical predictors for the development of cardiotoxicity [15, 16]. This is most likely due to our small sample size. Pilot studies have shown the great potential of a combination of echocardiographic screening and various biomarkers [17, 18]. Future research should be directed at optimising identification of the patients at highest risk of developing cardiotoxicity, who deserve intensified cardiac follow-up, in order to warrant the most efficient screening.

In our study, once subclinical 3D LVEF deterioration occurred, trastuzumab was paused and heart failure therapy was initiated empirically on an individualised basis by the treating cardiologist. Whether initiation of cardioprotective medications improves the outcome in patients with subclinical cardiac dysfunction is not entirely clear. A study by Boekhout et al. did not support the hypothesis that concomitant use of candesartan protects against a decrease in LVEF during or shortly after trastuzumab treatment in breast cancer patients [19]. In the MANTICORE trial, 94 patients were randomised to either placebo, perindopril or bisoprolol to investigate the cardioprotective effect. The authors showed signs of myocardial inflammation early after trastuzumab initiation. Patients on bisoprolol showed no deterioration of GLS after the first cycles in contrast to the other two groups. Both bisoprolol and perindopril were associated with preserved LVEF after completion of trastuzumab treatment [20]. In another trial, 468 trastuzumab-treated patients were randomised to either angiotensin-converting enzyme (ACE) inhibitors or beta-blockers or placebo. The authors found fewer interruptions of trastuzumab treatment in the first two groups, and the therapy prevented cardiotoxicity [21].

An ongoing larger randomised controlled trial with angiotensin-receptor inhibitors and beta-blockers in patients receiving anthracycline and trastuzumab and evaluated with strain measurements is still awaited [22]. The SUCCOUR trial, including 331 anthracycline-treated patients, showed that GLS-guided initiation of cardioprotective medication (ACE inhibitors and beta-blockers) may lead to a smaller reduction in LVEF after 1 year, in patients who received this cardioprotective treatment [7]. We demonstrated that, in all patients with cardiotoxicity in our study, 3D LVEF and GLS did recover after temporarily pausing trastuzumab and/or starting cardioprotective medication, even when trastuzumab was continued after a short pause. These are preliminary observations, which require longer-term follow-up. Some evidence indicates that, several years after chemotherapy, GLS values in asymptomatic cancer survivors remain significantly lower than in the overall population [23].

While the clinical consequence of subclinical cardiotoxicity remains uncertain, interrupting cancer therapy might have deleterious effects with regard to cancer outcome. Two studies aimed to determine whether trastuzumab may be continued despite a subclinical LVEF decline. One small but interesting prospective study suggested that, in patients administered ACE inhibitors and beta-blockers, trastuzumab may be continued safely despite mild cardiotoxicity [24]. However, two patients (10%) developed heart failure with an LVEF < 40%, but completely recovered after discontinuation of trastuzumab. Further randomised controlled trials are required to confirm this outcome in low-risk patients.

The SAFE-HEaRt trial studied LVEF during trastuzumab treatment in patients with an LVEF of 40%–49% prior to trastuzumab initiation [25]. They were treated with beta-blockers and/or ACE inhibitors. In 27 patients, trastuzumab treatment could be completed. However, trastuzumab was discontinued in 3 patients because of heart failure or an LVEF of < 35%. Two of them died due to disease progression. It is unknown whether the cardiac outcome outweighs the cancer outcome with respect to trastuzumab discontinuation.

### Limitations

While follow-up was relatively long, with multiple echocardiograms per patient, cardiotoxicity can occur in a later stage of life. Therefore, observations might change in either direction during a longer follow-up period. The study was limited by its retrospective nature and relatively small sample size. The latter may also explain the relatively weak correlation of GLS and 3D LVEF, although the correlation was found to be significant, as expected. Larger-scale studies are necessary particularly to identify the clinical risk factors for cardiotoxicity, thereby identifying the high-risk patients who need intensified cardiac follow-up. Although GLS is a very promising early detector of subclinical LV dysfunction, it does have some limitations. Currently, there are different echocardiography machines and software packages with potential intervendor variability of strain measurements [26]. Nevertheless, in 2015 an EACVI/ASE/Industry Task Force consensus document was published to standardise deformation imaging [10]. The reliability of GLS depends highly on the quality of the acoustic windows. Left-sided mastectomy or lumpectomy and breast reconstruction in this population of breast cancer patients pose a common problem in this respect.

## Conclusion

Myocardial strain imaging using GLS is able to identify subclinical LV dysfunction earlier than 3D LVEF measurement in women undergoing treatment with anthracyclines followed by trastuzumab for breast cancer. After pausing trastuzumab and starting cardioprotective therapy, this LV dysfunction is potentially reversible. It remains to be established whether the use of GLS to guide early cardioprotective therapy results in improved clinical outcomes and which patients are at highest risk of developing cardiotoxicity.

## Supplementary Information

Fig. S1

Fig. S2

Fig. S3

Fig. S4

Table 1. Baseline characteristics. Fig. S1 Kaplan Meier curve of longitudinal evolution of global longitudinal strain stratified to CRS scores of 5 and 7. *n* = 51. Fig. S2 Kaplan Meier curve of longitudinal evolution of global longitudinal strain over time in patients who did not reach the endpoint. *n* = 51. Fig. S3 Relative change in global longitudinal strain and absolute change in left ventricular ejection fraction compared to baseline in the 9 patients that reached the primary endpoint. Fig. S4 3D plot of prior global longitudinal strain (x‑axis) predicting left ventricular ejection fraction (y‑axis) on the following echocardiogram adjusted for time (z‑axis). Both an increase in time and worsening prior global longitudinal strain are associated with lower left ventricular ejection fraction. *n* = 51
