# Supplementary figures and images for: Global longitudinal strain: an early marker for cardiotoxicity in patients treated for breast cancer
Source: Neth Heart J. 2022 Nov 26;31(3):103–8. doi: 10.1007/s12471-022-01734-3 (PMC9950304; doi:10.1007/s12471-022-01734-3)

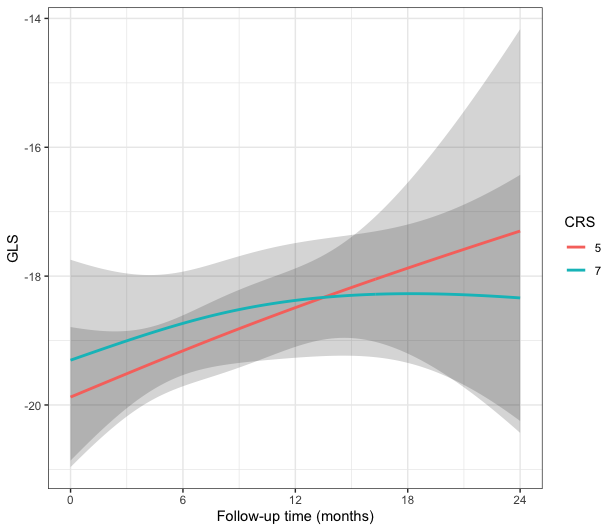

Supplement: Supplementary file 1 — Fig. S1 [file 12471_2022_1734_MOESM1_ESM.tiff]

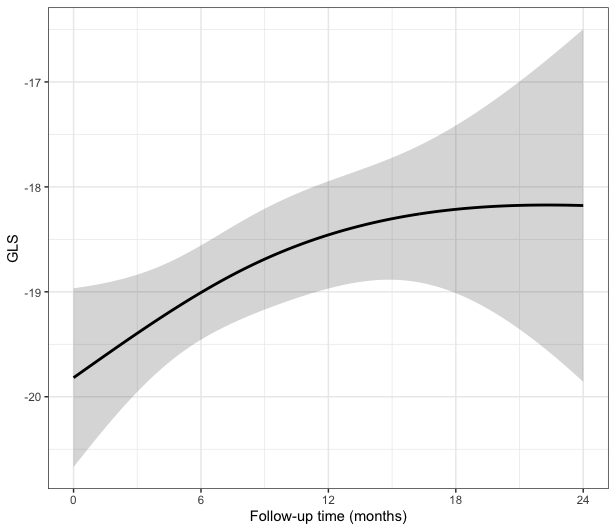

Supplement: Supplementary file 2 — Fig. S2 [file 12471_2022_1734_MOESM2_ESM.tiff]

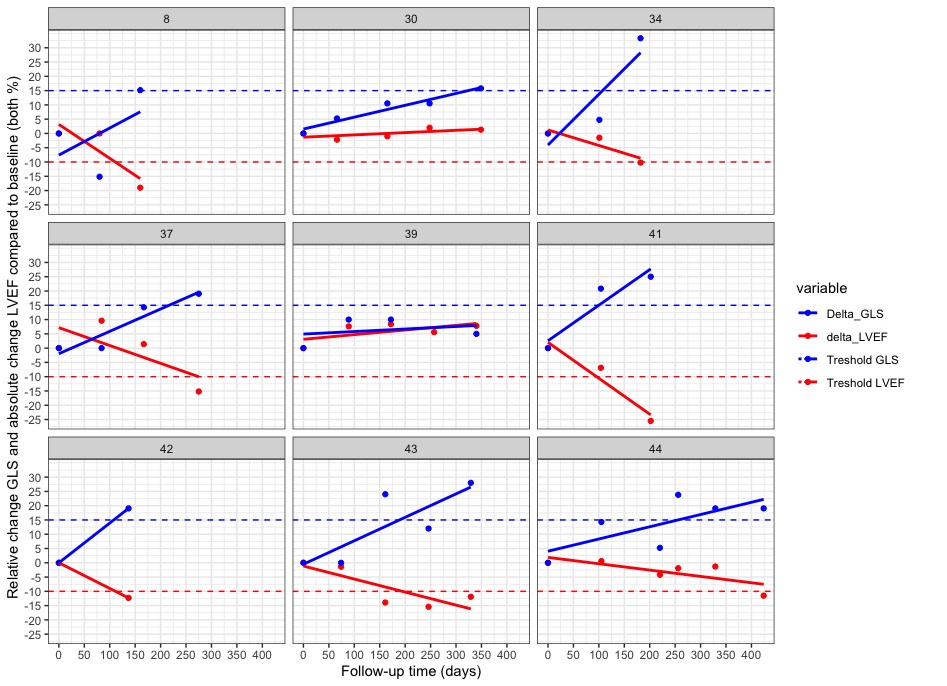

Supplement: Supplementary file 3 — Fig. S3 [file 12471_2022_1734_MOESM3_ESM.jpg]

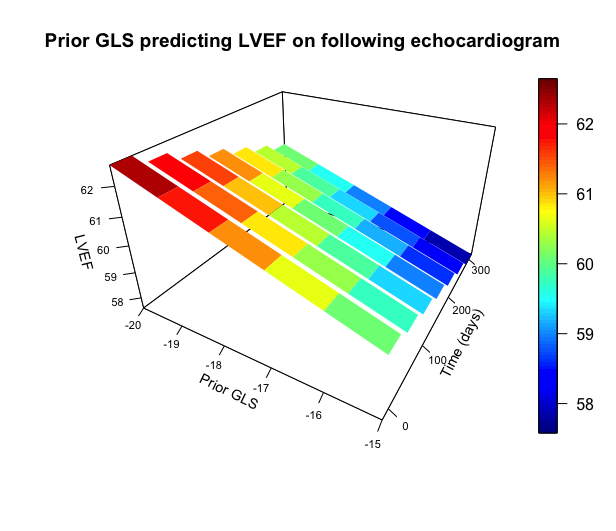

Supplement: Supplementary file 4 — Fig. S4 [file 12471_2022_1734_MOESM4_ESM.tiff]
