# Supplementary material for: Global longitudinal strain: an early marker for cardiotoxicity in patients treated for breast cancer
Source: Neth Heart J. 2022 Nov 26;31(3):103–8. doi: 10.1007/s12471-022-01734-3 (PMC9950304; doi:10.1007/s12471-022-01734-3)
Supplement: Supplementary file 5 — Table 1. Baseline characteristics. Fig. S1 Kaplan Meier curve of longitudinal evolution of global longitudinal strain stratified to CRS scores of 5 and 7. n = 51. Fig. S2 Kaplan Meier curve of longitudinal evolution of global longitudinal strain over time in patients who did not reach the endpoint. n = 51. Fig. S3 Relative change in global longitudinal strain and absolute change in left ventricular ejection fraction compared to baseline in the 9 patients that reached the primary endpoint. Fig. S4 3D plot of prior global longitudinal strain (x‑axis) predicting left ventricular ejection fraction (y‑axis) on the following echocardiogram adjusted for time (z‑axis). Both an increase in time and worsening prior global longitudinal strain are associated with lower left ventricular ejection fraction. n = 51 [file 12471_2022_1734_MOESM5_ESM.docx]

**Table S1** Baseline characteristics

| *n*=51 | Mean or median | 95% CI | IQR |
| --- | --- | --- | --- |
| Age | 54.0 | (50.5 - 57.6) |  |
| Length | 168.3 | (166.0 - 170.6) |  |
| Weight | 68.0 |  | (62.0 - 81.0) |
| BMI | 24.7 |  | (21.8 - 28.0) |
| BSA | 1.8 |  | (1.7 - 1.9) |
| Systolic blood pressure | 129.1 | (123.9 - 134.2) |  |
| Diastolic blood pressure | 71.0 |  | (66.0 - 80.0) |
| Haemoglobin | 8.0 | (7.8 - 8.3) |  |
| Creatinine | 67.3 | (63.8 -70.7) |  |
| eGFR | 88.0 |  | (73.0 - 90.0) |
|  |  |  |  |
|  | Frequency | Percentage |  |
| History of coronary artery disease | 2 | 3.9% |  |
| Other cardiac history | 3 | 5.9% |  |
| Diabetes mellitus | 3 | 5.9% |  |
| Hypertension | 10 | 19.6% |  |
| Smoking current | 5 | 9.8% |  |
| Smoking former | 7 | 15.6% |  |
| Peripheral artery disease | 1 | 2.0% |  |
| Minor stroke | 2 | 3.9% |  |
| Cardiovascular family history | 8 | 22.2% |  |
| Cardiovascular medications: |  |  |  |
| ACE inhibitor | 3 | 5.9% |  |
| Beta-blocker | 6 | 11.8% |  |
| ARB | 4 | 7.8% |  |
| Diuretic | 6 | 11.8% |  |
| Statin | 3 | 5.9% |  |
| No cardiac medication | 38 | 74.5% |  |
|  |  |  |  |
| Previous oncological treatments | 4 | 11.8% |  |
| Previous anthracycline | 2 | 3.9% |  |
| Previous trastuzumab | 1 | 2.0% |  |
| Previous radiotherapy | 2 | 3.9% |  |
| Cardiotoxicity risk score |  |  |  |
| 5 | 33 | 64.7% |  |
| 6 | 10 | 19.6% |  |
| 7 | 5 | 9.8% |  |
| 8 | 2 | 3.9% |  |
| 9 | 1 | 2.0% |  |

**Figure legends for supplementary figures**

**Fig. S1** Kaplan Meier curve of longitudinal evolution of 3D left ventricular ejection fraction stratified to CRS scores of 5 and 7. *n*=51

**Fig. S2** Kaplan Meier curve of longitudinal evolution of global longitudinal strain stratified to CRS scores of 5 and 7. *n*=51

**Fig. S3** Relative change in global longitudinal strain and absolute change in left ventricular ejection fraction compared to baseline in the 9 patients that reached the primary endpoint.

**Fig- S4** 3D plot of prior global longitudinal strain (x-axis) predicting left ventricular ejection fraction (*y*-axis) on the following echocardiogram adjusted for time (*z*-axis). Both an increase in time and worsening prior global longitudinal strain are associated with lower left ventricular ejection fraction. *n*=51.
